# Measurement and Characterization of Electromagnetic Noise in Edge Computing Networks for the Industrial Internet of Things

**DOI:** 10.3390/s19143104

**Published:** 2019-07-13

**Authors:** Huiting Li, Liu Liu, Yiqian Li, Ze Yuan, Kun Zhang

**Affiliations:** School of Electronic and Information Engineering, Beijing Jiaotong University, Beijing 100044, China

**Keywords:** edge computing, Industrial Internet of Things, electromagnetic noise, measurement, continuous hidden Markov model

## Abstract

Edge computing and the Internet of Things (IOT) provide the technological basis for the development of intelligent manufacturing nowadays. In order to support the intelligent interconnection and application of all kinds of equipment in the industrial field, edge computing should be equipped close to or embedded in all kinds of equipment nodes in the industrial wireless network. Therefore, it is meaningful to investigate the wireless network design of the Industrial Internet of Things. Low power wireless sensor devices are widely used in the Industrial Internet of Things (IIoT), which are sensitive to electromagnetic noise. The electromagnetic noises in industrial scenarios are significantly different from the conventional assumed white noise. In this paper, the measurement results of electromagnetic noises at three different test positions are given in an automobile factory. The spectrum occupancy of the factory wireless environment in the 300 MHz–3 GHz band was obtained by frequency domain measurement. In the time domain measurement, four statistical parameters of the three bands of 315 MHz, 433 MHz, and 916 MHz were measured, and the electromagnetic noise distributions in different plant areas and different frequency bands were analyzed. According to the measurement results, the time-varying characteristics of electromagnetic noise can be characterized by continuous hidden Markov models (CHMM). These results are informative to the design and optimization for the edge computing networks for IIoT.

## 1. Introduction

With the proposal of the industrial 4.0 strategy, advanced technologies such as the Internet of Things, edge computing, and Big Data have emerged to provide technical support for the development of intelligent manufacturing [1,2,3,4]. The Industrial Internet of Things connects machines, objects, and people in factories [5,6]. Edge computing transfers data processing, application running, and even the realization of some functional services from the network center to the nodes on the edge of the network. It solves the disadvantage that cloud computing cannot support computing-intensive and delay-sensitive tasks and enables data processing and analysis in intelligent factories. In order to support the intelligent interconnection and application of all kinds of equipment in the industrial field more effectively, edge calculation should be made close to or embedded in all kinds of sensor, robot, instrument, and other equipment nodes of industrial wireless networks [7]. In order to better access or embed edge computing on various device nodes of industrial wireless networks, the characteristics of the Industrial Internet of Things should be studied.

As a typical case of the fifth generation mobile communication (5G) mMTC (massive machine type of communication) scenario, the Industrial Internet of Things (IIoT) has become the key competitiveness of the country and has attracted extensive attention in academic and industrial fields [8]. Wireless technology, as the information transmission channel in the IIoT system, plays a significant role in the industrial field. At present, improving production efficiency and realizing energy saving and emission reduction are the main strategic tasks of manufacturing industry. With the development of the industrial internet, intelligent manufacturing will run through all aspects of enterprise production and operation, bringing profound changes to the development of manufacturing industry. IIoT connects mechanical equipment, people, and items together and enables them to exchange data. It will be widely used in automobile manufacturing and other industrial scenarios. However, the electromagnetic noise in the industrial environment is significantly different from that in other typical environments such as office areas [9]. When the machine is working, different types of electromagnetic noises will be emitted due to the increase of temperature, mechanical vibration, spark discharge, and other physical phenomena. Moreover, there are many kinds of electromagnetic noise distributed at any random position in the factory, and the frequency distribution range is very large [10]. Electromagnetic noise causes electromagnetic interference to the industrial environment, which reduces the signal-to-noise ratio of the communication system and affect the communication quality and the capacity of the communication system.

Noise is usually considered as additive white Gaussian noise in general scenes. However, the noise characteristics in IIoT scenes are quite different from the additive white Gaussian noise (AWGN) hypothesis [11]. First, the noise generated by machines, motors, and other equipment may have a non-flat power spectrum, so the noise in the IIoT scene does not always meet the characteristics of white noise [12]. Second, the frequency domain impulse noise generated by welding equipment in infrared environments does not obey the time domain Gaussian distribution, so the noise in IIoT scenes does not always obey the Gaussian distribution. Third, the noise process in IIoT scenes is not always stationary, and there often exist sudden noises. In addition, in order to prolong the service life, equipment in industrial applications is in a low power transmission mode, which makes them vulnerable to industrial noise generated by machines, motors, and other equipment [13]. Therefore, it is meaningful to study the wireless network design of the Industrial Internet of Things.

At present, previous scholars had carried out research on electromagnetic noise in the factory environment. In [14], the first-order Markov process was used to model noise in the factory environment, and noise in the factory was compared with additive white Gaussian noise. The analysis results showed that factory noise was not a conventional additive white Gaussian noise, and the properties between them were significantly different. However, the difference between noise in factories and additive white Gaussian noise was only discussed theoretically in this paper, and the actual measurement was not considered. In [15], the time domain measurement of noise was carried out in paper mills and iron and steel mills by using a spectrum analyzer, and the amplitude probability distribution (APD) information of noise at 439 MHz, 440 MHz, 570 MHz, and 2450 MHz was provided. However, the time-varying characteristics of noise were not discussed in this paper. In [16], the researchers measured the impulse noise generated by an automotive ignition system and an automotive electronic parts manufacturing system at 2.4 GHz frequency point, but the typical frequency bands of the IIoT system such as 315 MHz, 779 MHz, and other possible frequencies were not considered.

In this paper, log-period antennas and spectrum analyzers were used as measurement and analysis equipment. Based on the measurement results in the frequency domain, the frequency occupation in the factory environment was analyzed. According to the time domain measurement results at 315 MHZ, 779 MHz, and 916 MHZ, the amplitude probability distribution (APD), noise amplitude distribution (NAD), pulse duration distribution (PDD), and pulse separation distribution (PSD) of noise were extracted, and the characteristics of electromagnetic noise were investigated. Finally, the continuous hidden Markov models (CHMM) were used to model the time-varying characteristics of electromagnetic noise.

The rest of this article is organized as follows. The descriptions of the measurement environment and methods are provided in Section 2. In Section 3, we use four types of parameter extraction methods, APD, NAD, PDD, and PSD, to analyze the statistical properties of the gathered noises. The results of noise measurement in the frequency domain and time domain are given in Section 4 and Section 5, respectively. In Section 6, a brief conclusion is given.

## 2. Measurement Environments and Methods

### 2.1. Measurement Positions

**Figure 1:**
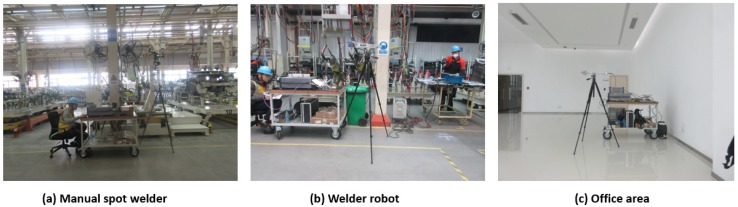
Real scenes of the three positions. (**a**) Manual spot welder; (**b**) Welder robot; (**c**) Office area.

The measurements were conducted in an automobile factory. The automobile factory has two main areas; one is the processing area and the other is the office area. Welding is the most important manufacturing process in the processing area, including the manual spot welder and the welder robot. We selected three positions for the measurements: near the manual spot welder, near the welder robot, and in the office area. The real environments of the three positions are shown in Figure 1. In the process of measurement, the equipment was at work. Compared with the welding workshop, the office area can be seen as a noise-free area, and the measurement results in there can be used for comparison.

### 2.2. Measurement Equipment

A logarithmic periodic antenna and spectrum analyzer were used for noise measurement. The logarithmic periodic antenna is commercial and has many outstanding advantages, such as wider operating bandwidth and superior gain for all frequencies in the frequency band range. The operating frequency range of this antenna is 300 MHz to 8 GHz. The size of the antenna is 355×497 mm, and the polarization mode is linear polarization. In actual measurement, the antenna was elevated to 1.4 m high. The antenna was measured in horizontal and vertical polarization modes.

**Figure 2:**
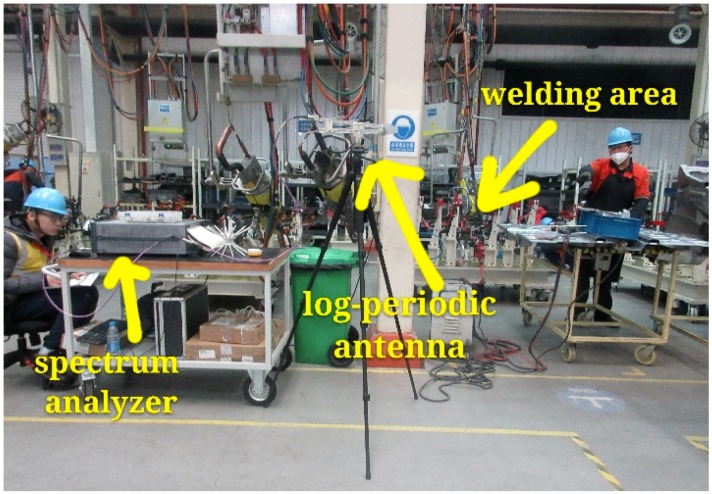
Actual scene of the measurement.

We conducted the measurements in frequency and time domains. The frequency domain measurement was done to analyze the occupancy of radio spectrum resources. We extracted the central frequency band, bandwidth, and power level of the noise. By using the time domain measurement, the time waveform of the electromagnetic noise was recorded at certain frequency bands. We analyzed the statistical properties in the time domain. The actual measurement environments are shown in Figure 2.

### 2.3. Frequency Domain Measurement Method

The spectrum analyzer used was the N9010B spectrum analyzer manufactured by Keysight Company, and its measurement range was 10 Hz–44 GHz. In measurement, the parameter setting of the spectrometer has a great influence on the accuracy of test results, so reasonable parameter setting is very important. The occupied BW (bandwidth) mode of the spectrum analyzer was used in the frequency domain measurement method. We measured the frequency occupation from 300 MHz to 3 GHz at these three positions. The horizontal polarization and vertical polarization of the antenna were measured, respectively. The preamp was set to the open state. The parameters of frequency domain measurements are listed in Table 1.

### 2.4. Time Domain Measurement Method

The data collecting mode of the spectrum analyzer was used in the time domain measurement. We recorded the time domain waveform of electromagnetic noise at 315 MHz, 779 MHz, and 916 MHz. These three frequencies and the 200 kHz bandwidth selected by the time domain measurement are the frequency points and bandwidth adopted by low power wireless sensor chips on the market. The sampling rate was set to 2 MHz and the measurement duration was set to 134.2 s. As in frequency domain measurement, the antenna was measured by horizontal polarization and vertical polarization, respectively. The parameters of time domain measurements are listed in Table 2.

## 3. Statistical Parameters

**Figure 3:**
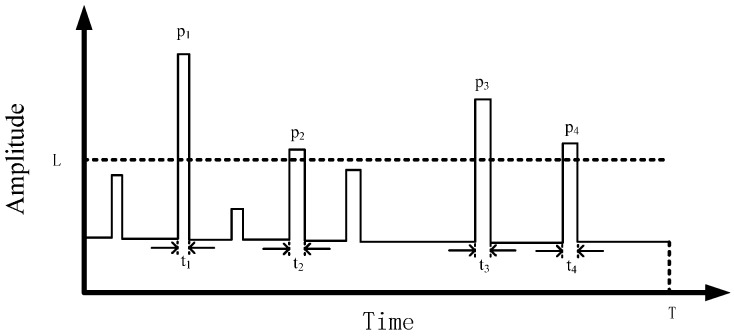
A simple envelope model.

As seen in Figure 3, a simple noise baseband model was plotted [17]. Three important characteristics of the electromagnetic noise baseband model were considered: the amplitude of the pulse, the duration of the pulse, and the interval between the two adjacent pulses. Four statistical parameters were employed to analyze the three characteristics of the noise spectrum [17]: amplitude probability distribution (APD), pulse duration distribution (PDD), pulse separation distribution (PSD), and noise amplitude distribution (NAD). The APD and NAD were used to study the amplitude characteristics of pulses, PDD was used to analyze the duration of pulses, and PSD could well characterize the arrival interval of adjacent pulses.

The APD and NAD were used to characterize the amplitude of the pulse. The APD represents the probability that the pulse amplitude exceeds a certain threshold value. It can be calculated by the ratio of the total time when the pulse amplitude exceeds the threshold value to the total time measured as [18].

(1) $$APD(L_{i})=P(\lambda >L_{i})=\frac{1}{T}\sum_{k=1}^{N}t_{k}$$

where λ is the noise amplitude and $L_{i}$ is the threshold level; we refer to the method proposed in patent [19] to determine it in the actual measurement and analysis process. T is the total measurement time, *t_k_* is the duration of a pulse whose amplitude exceeds the fixed value. For instance, the APD of the signal in Figure 3 is $APD(L)=P(\lambda >L)=(t_{1}+t_{2}+t_{3}+t_{4})/T$. The influence of electromagnetic noise on bit error rate (BER) can be mapped directly to the APD value, which can directly reflect the size of the BER.

The NAD is defined as the number of pulses whose value is higher than the threshold divided by the total measurement time [20,21], and is expressed as

(2) $$NAD(L_{i})=\frac{n(p_{K})}{T}$$

where T is the total measurement time and $n(p_{k})$ is the number of pulses whose value is higher than the threshold. For instance, the NAD of the signal in Figure 3 is $NAD(L)=n(p_{K})/T=4/T$.

The PDD is used to analyze pulse duration. It means the probability that the duration of the pulse exceeds a threshold value $\tau_{d}$, which can be expressed by the ratio of the number of pulses lasting time beyond $\tau_{d}$ to the total number of pulses as [17]:

(3) $$PDD\left(\tau_{d}\right)=\frac{n(t>\tau_{d})}{N}$$

where $n(t>\tau_{d})$ is the number of pulses lasting time beyond $\tau_{d}$ and N is the total number of pulses.

PSD can well characterize the arrival interval of adjacent pulses. It means the probability that the interval of adjacent pulses exceeds a fixed value $\tau_{s}$ [16]. It can be expressed as

(4) $$PSD\left(\tau_{S}\right)=P(t>\tau_{s})$$

where $P(t>\tau_{s})$ is the probability that the duration of the pulse exceeds the fixed value.

According to the above theory, the time domain electromagnetic noise signals at 315 MHz, 779 MHz, and 916 MHz frequencies were measured, and the APD, PDD, PSD, and NAD statistical parameters of electromagnetic noises at different frequencies could be extracted.

## 4. Characterization Using the Frequency Domain Measurement Data

**Figure 4:**
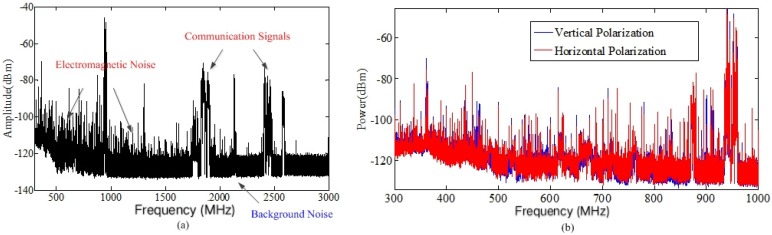
Spectrums of the noise measured near welder robot. (**a**) Amplitude spectrum of noise; (**b**) Power spectrum of noise.

The measurement results of the welder robot are shown in Figure 4. As shown in Figure 4a, the received signals contained electromagnetic noise, background noise, and operating mobile communication signals, such as 2G (Global System for Mobile Communications, GSM) and 4G (Long Term Evolution, LTE) signals. Table 3 shows the spectrum occupancy rate of existing communication signals, in which the operating mobile communication signals were mainly between 900 MHz and 1800 MHz. In Figure 4a, we can see that mobile communication signals had wideband spectrums and high power levels. However, the power of electromagnetic noise was small, mainly below 1 GHz. Figure 4b shows that the results measured by horizontal and vertical polarization of the antenna were similar.

**Figure 5:**
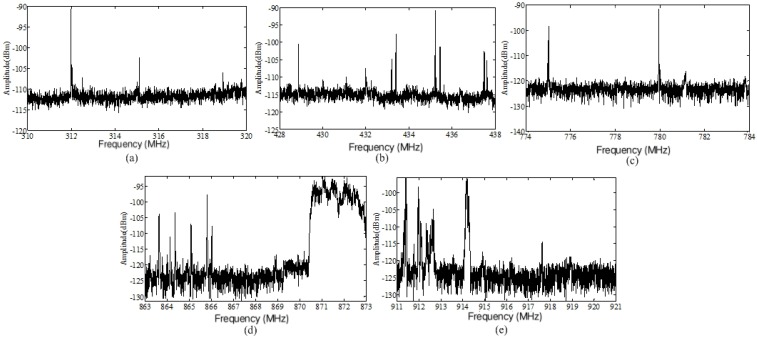
Spectrums of noises measured at five frequency bands. (**a**) Amplitude spectrum of noise at 315 MHz; (**b**) Amplitude spectrum of noise at 433 MHz; (**c**) Amplitude spectrum of noise at 779 MHz; (**d**) Amplitude spectrum of noise at 868 MHz; (**e**) Amplitude spectrum of noise at 916 MHz; (**f**) Amplitude spectrum of noise at 916 MHz.

Low power wireless sensors are often used in industrial scenarios. The noise power has a great impact on the wireless links of sensors. Low power wireless sensors usually work in five frequency bands, i.e., 315 MHz, 433 MHz, 779 MHz, 868 MHz, and 916 MHz, and the bandwidth is usually from 10 kHz to 200 kHz. Therefore, we focused our measurements on the spectral bands near the above five bands. Figure 5 shows the spectrum near the above frequency bands. Note that there were mobile communication signals in the frequency bands above 870 MHz and below 915 MHz. We extracted the average power levels of five bands of noise, as shown in Table 4. According to the extracted results, we could see that the power of the electromagnetic noise was decreased with the increase of the frequency.

However, according to the principle of the transmission theory, the path loss increases with the increase of frequency. The frequency selection of the IIoT needs to consider not only noise but also path loss. Therefore, we needed to calculate the path loss in different frequency bands. Thus, we could judge which frequency band is most suitable for IIoT.

The expression of path loss in free space is:

(5) L(d)=32.45+20lg(f)+20lg(d)

where f is the frequency in MHz and d is the distance between the transmitter and the receiver. The unit is kilometers. We assumed that d was equal to 0.1 km. We calculated the path loss according to Equation (5) and the results are shown in Table 5.

Taking 315 MHz as a reference, let its gain be 0. We defined the gain equal to the reduction of the average power of the noise minus the increase of the transmission loss. Taking 868 MHz as an example, the average noise power was reduced by 8.3 dB ((−111 dBm) − (−119.3 dBm)) compared to 315 MHz, and the path loss was increased by 8.8 dB (71.2 dB − 62.4 dB). Therefore, the gain of 916 MHz was −0.5 dB (8.3 dB − 8.8 dB). Similarly, we calculated the gain of other frequency bands. The specific results are listed in Table 6. According to the results in Table 6, the best choice of frequency of IIoT was 916 MHz, and the others were 315 MHz, 433 MHz, 868 MHz, and 779 MHz.

## 5. Characterization Using the Time Domain Measurement Data

### 5.1. Measurement Results

#### 5.1.1. APD Results

**Figure 6:**
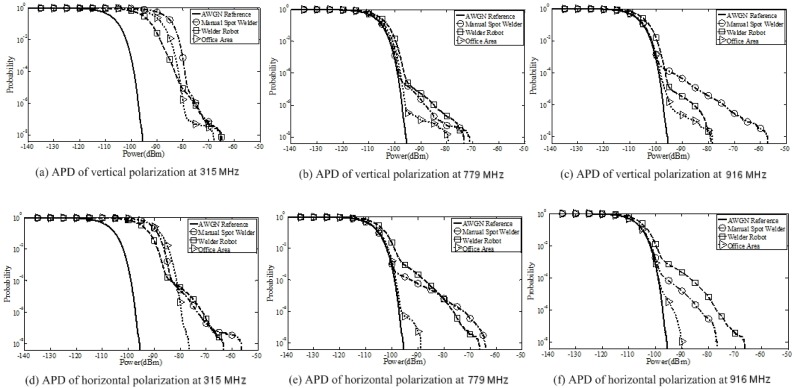
Amplitude probability distribution (APD) results. (**a**) APD of vertical polarization at 315 MHz; (**b**) APD of horizontal polarization at 315 MHz; (**c**) APD of vertical polarization at 779 MHz; (**d**) APD of horizontal polarization at 779 MHz; (**e**) APD of vertical polarization at 916 MHz; (**f**) APD of horizontal polarization at 916 MHz.

APD values of the measured noise are shown in Figure 6. For comparison, we also measured APD values of traditional additive white Gaussian noise, as shown in the solid line in Figure 6. Figure 6 shows that the APD values measured by the horizontal and vertical polarization modes of the antenna were approximately the same. The value of APD can be used as a measure of electromagnetic energy. The tail of the APD curve represents the maximum value of measured noise power. From the figure, we can see that the measured noise power values near the manual spot welder and the welder robot were similar, while the value measured in the office area was far less than that in the welding workshop, since the office area was less affected by mechanical equipment. The power of noise measured at the 916 MHz frequency point was the smallest, which was because the propagation loss increased with the increase of noise frequency.

#### 5.1.2. NAD Results

**Figure 7:**
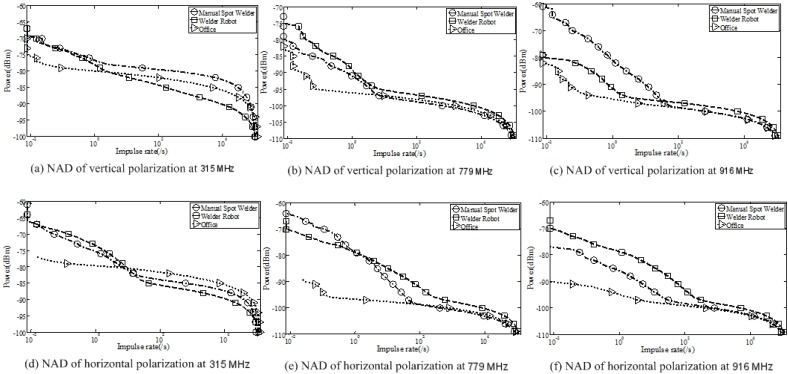
Noise amplitude distribution (NAD) results. (**a**) NAD of vertical polarization at 315 MHz; (**b**) NAD of horizontal polarization at 315 MHz; (**c**) NAD of vertical polarization at 779 MHz; (**d**) NAD of horizontal polarization at 779 MHz; (**e**) NAD of vertical polarization at 916 MHz; (**f**) NAD of horizontal polarization at 916 MHz.

NAD results of the measured noise are shown in Figure 7. The NAD is defined as the number of pulses whose value is higher than the threshold divided by the total measurement time. It measures the intensity of electromagnetic noise from another perspective. Figure 7 shows that the NAD values measured by the horizontal and vertical polarization modes of the antenna were approximate. The head (at the level of $10^{-2}$) of the NAD curve indicates that the power of electromagnetic noise measured in the office area was smaller than the power level measured in the manual spot welder and welder robot, which was due to the office area being less affected by electromagnetic noise. The NAD values of electromagnetic noise measured near the manual spot welder and the welder robot were similar, indicating that the intensity of electromagnetic noise near them was similar.

#### 5.1.3. PDD Results

**Figure 8:**
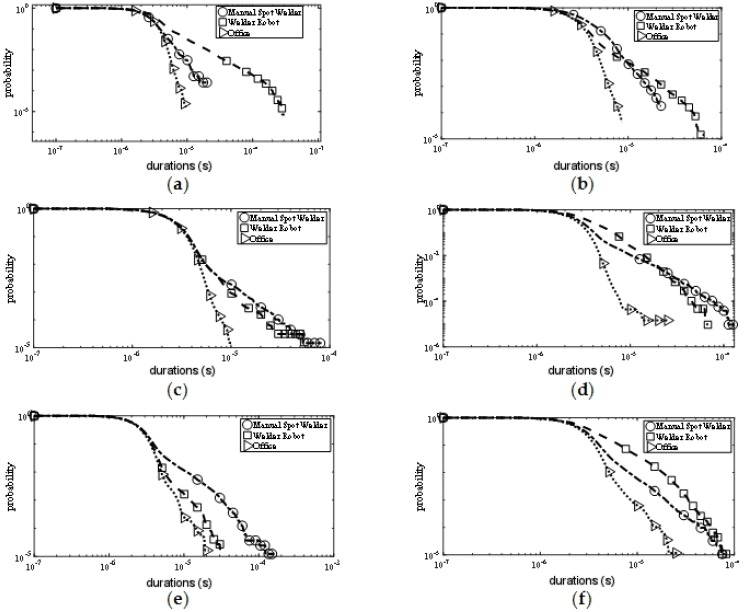
Pulse duration distribution (PDD) results. (**a**) PDD of vertical polarization at 315 MHz; (**b**) PDD of horizontal polarization at 315 MHz; (**c**) PDD of vertical polarization at 779 MHz; (**d**) PDD of horizontal polarization at 779 MHz; (**e**) PDD of vertical polarization at 916 MHz; (**f**) PDD of horizontal polarization at 916 MHz.

The PDD values of the noise are shown in Figure 8. PDD means the probability that the duration of the pulse exceeds a threshold value $\tau_{d}$. It can be seen from the measurement results that the PDD value of the office area was always smaller than the values at the other two measurement positions in the three frequency bands. This means that the duration of noise in the office area was the shortest during the same measurement time. The PDD values measured near the welder robot and the manual spot welder were not exactly the same, which may be due to the fact that the contact time between the welding head and the object was not a fixed value during manual welding, so the duration of pulse noise varied greatly.

#### 5.1.4. PSD Results

**Figure 9:**
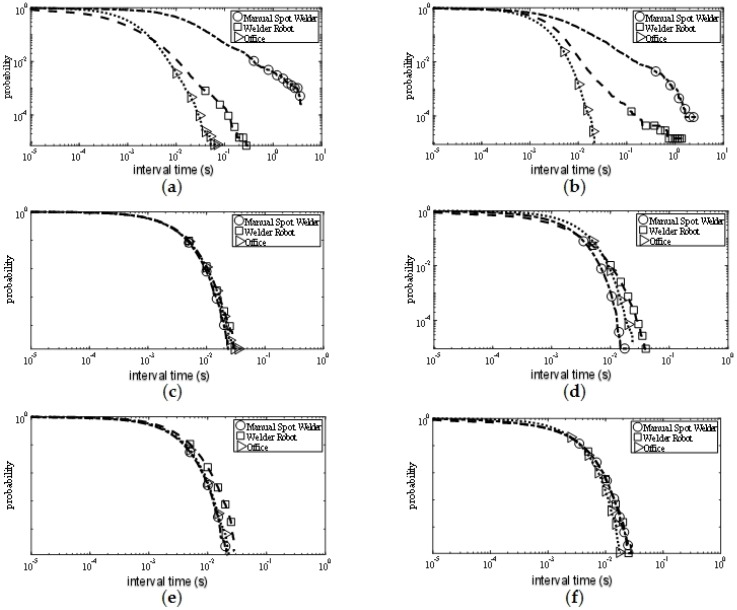
Pulse separation distribution (PSD) results. (**a**) PSD of vertical polarization at 315 MHz; (**b**) PSD of horizontal polarization at 315 MHz; (**c**) PSD of vertical polarization at 779 MHz; (**d**) PSD of horizontal polarization at 779 MHz; (**e**) PSD of vertical polarization at 916 MHz; (**f**) PSD of horizontal polarization at 916 MHz.

The PSD value of the noise is shown in Figure 9. PSD means the probability that the interval of adjacent pulses exceeds a fixed value $\tau_{s}$. From the measurement results, it can be seen that at the 315 MHz frequency point, the PSD value measured near the manual spot welder was higher than that measured near the welder robot, while the PSD value measured in the office area was the smallest. This shows that the noise power spectrum density value of the office area was smaller than the values of the other two measurement positions, while the uncertainty caused by manual operation caused electromagnetic noise to appear more frequently. In the 779 MHz and 916 MHz bands, the PSD values measured at the three locations were similar, which may be due to the frequency increase, and the noise power spectrum density values at the three measurement locations tended to be consistent.

Combining the values of the four statistical parameters shown in Figure 6, Figure 7, Figure 8 and Figure 9, it can be concluded that the electromagnetic noise generated by the manual spot welder and the welder robot operation was similar, but it was not completely consistent. This was because the two kinds of mechanical equipment used the same welding head, so the noise situation was similar. However, when working, the height of the welding head and the position of the welding car were different, resulting in slightly different noises, but the two were generally similar.

Since the tail of the APD curve represents the maximum measured noise power, it can be seen from the APD results that the maximum measured noise power in the office area was significantly lower than the maximum measured noise power in the other two measurement positions under the same frequency point and the same antenna polarization mode. PDD results indicate that the office area was less affected by electromagnetic noise with a longer duration than the other two measurement positions. PSD results indicate that the pulse interval of electromagnetic noise in the office area was larger than the other two measurement positions. From these three data, it can be seen that the office area was less affected by electromagnetic noise than the other two measurement positions, which was due to the fact that both manual spot welding machines and welding machines were located inside the welding workshop, and the workshop environment was more noisy and electromagnetic interference was stronger than that of the office area. However, the office area was not far from the welding workshop and also had interference by electromagnetic noise radiated from the workshop, so the noise power measured in the office area was only slightly lower than that measured in the welding workshop.

In addition, we can conclude from the NAD results that an electromagnetic noise higher than −90 dBm was generated at every 0.1 ms interval at 315 MHz near the welding robot. However, the received power of the user equipment in the IIOT scenario was about −90 dB. Therefore, these electromagnetic noises will greatly affect the reliability of the communication system. Therefore, it is necessary to further study the anti-noise problem in industrial environments.

### 5.2. Modeling of Time-Varying Characteristics

In the welding shop, electromagnetic noise occurs and disappears due to the work conditions of the mechanical equipment. The continuous hidden Markov model is a commonly used method for statistical modeling of non-stationary signals, such as speech signals. We used the continuous hidden Markov model (CHMM) to characterize the time-varying characteristics of the electromagnetic noise.

We consider that there are two states (state 1 and state 2) of electromagnetic noise in the factory. State 1 and 2 represents the equipment is not working or is working, respectively. We assume that both states are Gaussian distributed variables and the mean and variance are $\mu_{1}$, $\sigma_{1}$, $\mu_{2}$, and $\sigma_{2}$, respectively. Thus, a two-state first-order CHMM can be used to characterize the generation and extinction behavior of the electromagnetic noise in industrial scenarios.

#### 5.2.1. CHMM Definition

CHMM has three main elements: state transition probability matrix P, state observation vector probability model B, and initial state probability vector I [22]. If the event has two states and they all obey the Gaussian distribution, then:

(6) $$P={\left[p_{ij}\right]}_{2\times 2}=\left[\begin{array}{cc} p_{11} & p_{12}\\ p_{21} & p_{22} \end{array}\right]$$

where $p_{ij}$ means the probability of making a transition to state j, given currently in state i.

(7) $$B=\{b_{1}(y),b_{2}(y)\}$$

where

(8) $$b_{1}(y)=\frac{1}{\sqrt{2\pi }\sigma_{1}}e^{-\frac{{\left(y-\mu_{1}\right)}^{2}}{2\sigma_{1}^{2}}},b_{2}(y)=\frac{1}{\sqrt{2\pi }\sigma_{2}}e^{-\frac{{\left(y-\mu_{2}\right)}^{2}}{2\sigma_{2}^{2}}}$$

$b_{1}(y)$, and $b_{2}(y)$ are the probability density function of two states.

(9) $$I=\left(I_{i}\right)=\left(I_{1},I_{2}\right)$$

where $I_{i}$ is the probability of the initial moment of state *i*.

To model the signal with CHMM (λ=(P,B,I)), we give an approximate estimate value of the three elements according to the real measurement data using the Baum–Welch algorithm [23]. Then the CHMM is used to simulate the non-stationary conditions of the electromagnetic noise.

#### 5.2.2. CHMM Modeling Method

**Figure 10:**
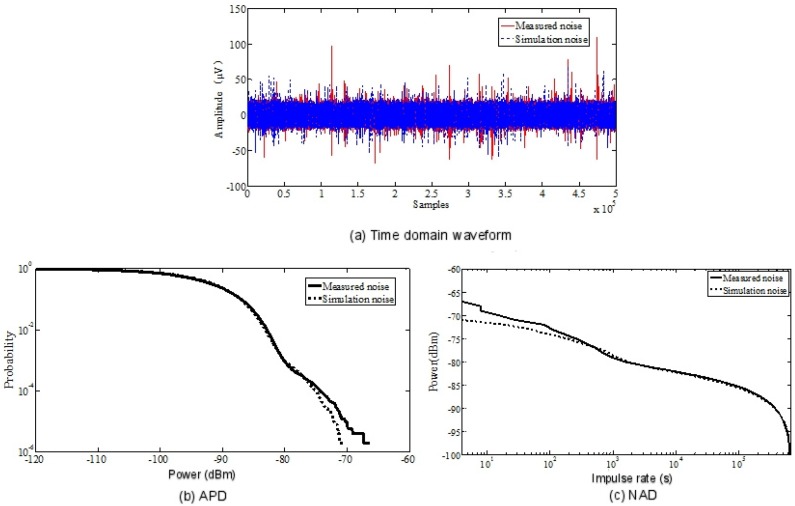
Comparison of measurement and simulation noise. (**a**) Time domain waveform; (**b**) Amplitude probability distribution (APD) value comparison of simulation noise and measurement noise; (**c**) Noise amplitude distribution (NAD) value comparison of simulation noise and measurement noise.

The solid red line in Figure 10a shows the time domain waveform of the electromagnetic noise measured at 315 MHz. First, an approximate estimate of the three elements for the λ is given. According to the measured data, the estimated values of P and I are:

(10) $$P=\left[\begin{array}{cc} 0.995 & 0.005\\ 0.1 & 0.9 \end{array}\right]$$

(11) $$I={\left(0.9,0.1\right)}^{T}$$

To make a rough estimate of the value of B, it is actually to estimate the four values of $\mu_{1}$, $\mu_{2}$, $\sigma_{1}$, and $\sigma_{2}$. We select the threshold according to the method in [24] and take the mean and variance of the noise below and above the threshold as a rough estimate of $\mu_{1}$, $\sigma_{1}$, $\mu_{2}$, and $\sigma_{2}$. Based on the measurement data, the extracted results are $\mu_{1}=0.0019$, $\mu_{2}=2.5470$, $\sigma_{1}=5.9967$, and $\sigma_{2}=35.7637$.

Secondly, the Baum–Welch algorithm is used to get accurate values of the λ=(P,B,I). The accurate estimation results for elements of CHMM are:

(12) $$P=\left[\begin{array}{cc} 0.9993 & 0.0007\\ 0.1761 & 0.8239 \end{array}\right]$$

(13) $$I={\left(1,0\right)}^{T}$$

(14) $$b_{j}(y)=\frac{1}{\sqrt{2\pi }\sigma_{j}}e^{-\frac{{\left(y-\mu_{j}\right)}^{2}}{2\sigma_{j}^{2}}},\;j=1,2$$

where $\mu_{1}=0$, $\mu_{2}=0.8770$, $\sigma_{1}=5.9696$ and $\sigma_{2}=17.9763$.

By using the previous parameterized CHMM model, we can simulate the noise, and the waveform in the time domain is shown in Figure 10a.

As shown in Figure 10a, dotted lines represent simulated noise while the real lines represent measured noise. Figure 10b,c shows the APD and NAD values of the measured noise and the noise simulated by the CHMM model. It can be seen that the noise simulated by the CHMM model was very similar to the measured noise, whether APD or NAD values. Thus, the CHMM model can be used to characterize the time-varying characteristics of electromagnetic noise. In addition, it can be concluded from these discussions that the electromagnetic noise in industrial scenes is different from the additive white Gaussian noise. It will seriously affect the signal-to-noise ratio of wireless communication systems, thus affecting the quality of data transmission in the Industrial Internet of Things. Corresponding measures must be taken to reduce the interference of electromagnetic noise, usually using OFDM or interleaved coding technology.

## 6. Conclusions

In recent years, edge computing and the Internet of Things (IOT) have provided a technological basis for the development of intelligent manufacturing. In order to support the intelligent interconnection and application of all kinds of equipment in the industrial field, edge computing should be close to or embedded in all kinds of equipment nodes in the industrial wireless network. In order to better access or embed edge computing on various device nodes of industrial wireless networks, the channel characteristics of the Industrial Internet of Things (IIoT) should be studied first. Therefore, it is very meaningful to study the wireless network design of the IIoT.

In this paper, the measurement results of electromagnetic noise at three different test positions were given in an automobile factory. The spectrum occupancy of a factory wireless environment in the 300 MHz–3 GHz band was obtained by frequency domain measurement. By calculating the average power of path loss and electromagnetic noise in different frequency bands, the optimal frequency band for the IIoT is 916 MHz. In time domain measurements, we analyzed the distribution of electromagnetic noise in different plant areas and in different frequency bands by measuring four statistical parameters of 315 MHz, 433 MHz, and 916 MHz. According to the measurement results, we concluded that the time-varying characteristics of electromagnetic noise can be characterized by CHMM.

According to the research results, we suggest that under an IIOT scenario, 916 MHz is the best frequency band for the equipment. The 779 MHz band with the most serious noise interference should be avoided as much as possible. The focus of this paper is the noise characteristics in an IIOT scene and its modeling analysis. Therefore, the research in this paper has some limitations. In the following research, the authors will further explore the channel and noise characteristics in the IIOT scene, understand the IIOT scene more deeply, and try to find effective methods to reduce electromagnetic noise interference in the IIOT scene.

## Figures and Tables

**Table 1:** Parameters of frequency domain measurements.

Parameters	Values
Frequency Band	300 MHz–3 GHz
Mode	Occupied BW
Span	50 MHz
RBW(Resolution Bandwidth)	10 kHz
VBW(Video Bandwidth)	100 kHz
Attenuation	2 dB
Preamp	Open
Points	20001
Polarized	Vertically/Horizontally

**Table 2:** Parameters of time domain measurements.

Parameters	Values
Frequency	315 MHz, 779 MHz, 916 MHz
Bandwidth	200 kHz
Sampling Rate	2 MHz
Measurement Duration	134.2 s
Mode	Data Collecting
Polarized	Vertically/Horizontally

**Table 3:** Frequency spectrums occupancy of communication signals.

Frequency (MHz)	Signal Type	Frequency (MHz)	Signal Type
478–486	Radio and TV Signals	825–835	CDMA UL
518–526		870–880	CDMA DL
526–534		890–915	GSM800 UL
614–622		935–960	GSM800 DL
654–662		1710–1755	GSM1800 UL
662–670		1755–1785	FDD-LTE UL
670–678		1805–1840	GSM1800 DL
702–710		1840–1875	FDD-LTE DL
718–726		1885–1915	TD-LTE
758–766		2130–2145	WCDMA DL
766–774		2401–2481	WIFI
790–798		2575–2595	TD-LTE

**Table 4:** Average power at different frequencies.

Frequency (MHz)	315	433	779	868	916
Average power of noise (dBm)	−111.0	−113.5	−118.3	−119.3	−124.1

**Table 5:** Path loss at different frequencies.

Frequency (MHz)	315	433	779	868	916
Path Loss (dB)	62.4	65.2	70.3	71.2	71.7

**Table 6:** Gain at different frequencies.

Frequency (MHz)	315	433	779	868	916
Gain (dB)	0	−0.3	−0.6	−0.5	3.8
